# Impact of negative symptoms on healthcare resource utilization and associated costs in adult outpatients with schizophrenia: a population-based study

**DOI:** 10.1186/s12888-014-0225-8

**Published:** 2014-08-06

**Authors:** Antoni Sicras-Mainar, Jorge Maurino, Elena Ruiz-Beato, Ruth Navarro-Artieda

**Affiliations:** Badalona Serveis Assistencials SA, Badalona, Spain; Medical Department, Roche Farma SA, Eucalipto, 33, 28016 Madrid, Spain; Health Economics and Outcomes Research Unit, Roche Farma SA, Madrid, Spain; Department of Medical Information, Hospital Universitari Germans Trias i Pujol, Badalona, Spain

**Keywords:** Schizophrenia, Negative symptoms, Resource use, Healthcare costs, Electronic medical records

## Abstract

**Background:**

To evaluate the prevalence and impact of negative symptoms on healthcare resources utilization and costs in patients with schizophrenia.

**Methods:**

A retrospective study was conducted using electronic medical records from the health provider BSA (Badalona, Spain). All adult outpatients with a diagnosis of schizophrenia were followed for 12 months. Two study groups were defined by the presence or absence of negative symptoms based on the PANSS Negative Symptoms Factor (N1-N4, N6, G7 and G16). Healthcare (direct cost) and non-healthcare costs (work productivity losses) were described. An ANCOVA model was used for correction, p < 0.05.

**Results:**

One thousand one hundred and twenty patients were included in the study (mean age: 46.8 ± 13.8 years; male: 58.4%). One or more negative symptoms were present in 52.5% of patients (95% CI: 49.6-55.4%). The most frequent were passive/apathetic social withdrawal and emotional withdrawal (60.5% and 49.8%, respectively). Patients with negative symptoms showed a greater mean number of comorbid conditions and pharmacological treatments. The adjusted unit healthcare cost related to the presence/absence of negative symptoms was €2,190.80 and €1,787.60 and the healthcare cost was €2,085.00 and €1,659.10, respectively; (p < 0.001). Patients with negative symptoms used more healthcare resources, mainly derived from primary care. The presence of negative symptoms was associated with being male, dyslipidemia, obesity and arterial hypertension (OR = 1.7, 1.4, 1.4 and 1.2, respectively).

**Conclusions:**

Negative symptoms are highly prevalent in adult outpatients with schizophrenia with a relevant economic impact on the healthcare system.

## Background

Schizophrenia is one of the leading causes of chronic incapacity and has a devastating impact on personal, social, and economic aspects of life [1,2]. Negative symptoms such as affective flattening, poverty of speech, general lack of motivation, asociality, and impaired attention are a critical unmet need for successful management of schizophrenia [3]. Negative symptoms are relatively common with one recent study finding that 58% of stable outpatients treated with second-generation antipsychotic drugs had at least one negative symptom [4]. Although the introduction of second-generation antipsychotics was expected to lead to a breakthrough in the control of negative symptoms, evidence suggests that these treatments have only a modest impact. Thus, many patients are left with negative symptoms after their positive symptoms have been partially or completely controlled [5,6].

Negative symptoms account for much of the long-term morbidity and poor functional outcome of patients with schizophrenia [7]. Using data from the CATIE trial of chronic schizophrenia, Rabinowitz et al. found that baseline functioning and change in functioning were more strongly related to PANSS negative symptom domains than any of the other symptoms [7]. Because of the proven impact that negative symptoms have on patient well-being, functioning, and health-related quality of life, it is crucial that clinicians can detect and address these symptoms early [8–11].

Research on the costs of care for patients suffering from schizophrenia has increased during recent years and it has been shown that high-income countries such as Spain typically spend up to 3% of their total healthcare-expenditure on schizophrenia care [12,13]. Apart from often substantial direct costs of treatment, specially associated with hospitalization, the indirect costs associated with loss of work productivity, unemployment and impacts on the family can contribute more than half the overall costs of the disorder [12–15]. Few studies suggested that there would be a positive association between direct medical costs and negative symptoms [16,17].

The primary objective of this study was to evaluate the prevalence and impact of negative symptoms on the use of healthcare resources and costs using data from the Badalona population database.

## Methods

### Study design

This is a non-interventional, retrospective, cohort study using electronic medical records held by the healthcare provider Badalona Serveis Assistencials (BSA). The study population consisted of patients from six outpatient centres managed by BSA. They cover a population of 120,000 inhabitants in a predominantly industrial urban setting with a medium-low socioeconomic status. The study protocol was approved by the investigational review board of the Hospital Universitari Germans Trias i Pujol (Badalona, Spain).

### Study population

The study included all outpatients who required care in 2011 that met the following criteria: a) age ≥18 years, b) a diagnosis of schizophrenia according to the DSM-IV-TR criteria [18], c) being under antipsychotic treatment, d) inclusion in the long-term prescriptions program (with a record of daily dose, time interval and duration of each treatment administered), and e) guaranteed regular patient follow-up (presenting ≥2 healthcare records in the computer system). Subjects who had moved to other areas and in whom the symptoms could not be characterised were excluded. Patients were followed for 12 months.

### Identification of negative symptoms

The Positive and Negative Syndrome Scale (PANSS) is a 30-item scale designed to capture the degree of severity for many symptoms in schizophrenia. In 2009, a workshop format under the auspices of the International Society for Clinical Trials Methodology (ISCTM) defined a consensus statement on negative symptoms [19]. The workshop expressed preference for the PANSS negative factors derived from factor analyses over the original PANSS negative subscale. The original PANSS negative symptom subscale contains two items, “stereotyped thinking” and “difficulty in abstract thinking,” which are outside the currently recommended negative symptom domains. The PANSS Marder Negative Symptoms Factor does not include these items [20].

A challenge with our clinical data resources was that symptom scales are not routinely completed in mental health services. This study sought to obtain information on the presence or absence of the negative symptoms of interest from language used in the text fields of electronic health records. In a first stage, detailed case note review was carried out by the investigating team in order to identify relevant terminology. The seven items of the PANSS Marder Negative Symptoms Factor were used as a framework for characterizing these symptoms from the database text fields: blunted affect (N1), emotional withdrawal (N2), poor rapport (N3), passive/apathetic social withdrawal (N4), lack of spontaneity and conversation flow (N6), motor retardation (G7), and active social avoidance (G16) [20]. Finally, there was a manual search for text extraction. Negative symptoms were classified as present or absent.

### Demographic and comorbidity variables

The primary study variables were: age (continuous and by ranges), gender, time since schizophrenia onset as well as personal history taken from the International Classification of Primary Care (ICPC-2) [21]. The following characteristics were used to summarise general comorbidity variables for each patient: a) the Charlson Comorbidity Index (CCI; as an approximation to patient severity) [22], b) the number of chronic diseases (diagnoses) and c) the individual Case-mix Index, obtained from the Adjusted Clinical Groups (ACG; a classification system based on the consumption of healthcare resources) [23]. The ACG application provides resource utilization bands (RUBs), so each patient is included in one of the five mutually exclusive categories depending on overall morbidity (1: healthy or very low morbidity, 2: low morbidity, 3: moderate morbidity, 4: high morbidity, and 5: very high morbidity).

### Pharmacological treatments

Information regarding administered treatment was obtained according to the Anatomical Therapeutic Chemical Classification System (ATC): a) sedatives/hypnotic drugs, b) antipsychotic agents, c) antidepressants, and d) other central nervous system drugs [24].

### Healthcare resources and costs

Direct healthcare costs were those related to care activity (medical visits, diagnostic or therapeutic requests, outpatient medication) carried out by healthcare professionals, while indirect costs were those related to work productivity losses (temporary or permanent sick leave).

The cost system design was defined considering the characteristics of the organisation and the degree of development of the available information systems. Cost was expressed as mean cost per patient (cost/unit). The different concepts and their economic values are shown in Table 1 (year 2012). The different fees were obtained from analytical accounts, except medication and days off work. Prescriptions were quantified by retail price per pack at the time of prescription and days absent from work were quantified according to minimum wage [25]. This study did not contemplate non-healthcare direct costs, classified as “out-of-pocket” costs paid by the patient/family, as they are not recorded in the database.

**Table 1:** **Use of resources and unit costs**

**Healthcare resources**	**Unit costs (€)**
Medical visits	
Primary care	23.19
Emergency care	117.53
Specialized care	104.41
Supplementary tests	
Lab tests	22.3
Radiology tests	18.5
Other diagnostic tests	37.12
Pharmaceutical prescription*	retail price/pack

Source of healthcare resources: Badalona Serveis Assistencials analytical accounts. Values expressed in euros (year 2012). Retail price includes VAT. *Includes cost of antipsychotic drugs and concomitant medication (antidepressants, anxiolytics/sedatives and other central nervous system active drugs).

### Statistical analysis

A descriptive univariate statistical analysis was performed with mean values, standard deviation and 95% confidence intervals (CI). Normal data distribution was verified using a Kolmogorov-Smirnov test. The bivariate analysis included ANOVA, the chi-squared test, Pearson’s linear correlation, and comparison of means. A logistic regression analysis was performed to obtain the variables associated to patient profile (negative symptoms), with the Enter procedure (statistic: Wald). Outpatient costs were compared as recommended by Thompson and Barber, by analysis of covariance (ANCOVA) of: age, gender, RUBs, Charlson Comorbidity Index, and time since schizophrenia onset (procedure: estimation of marginal means; Bonferroni adjustment) [26]. The SPSSWIN program, version 17 was used and statistical significance was established for values of p < 0.05.

## Results

**Figure 1:**
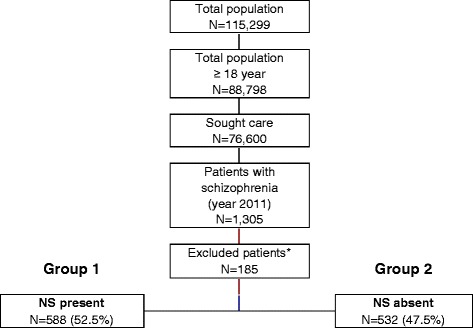
**General study disposition.** Follow-up for *all patients* was 1 year after inclusion. *Patients were excluded for the following: missing or inconsistent data (N = 33; 17.8%), loss to follow-up (N = 45; 24.3%), moved to other areas (N = 37; 20.0%) and other reasons (N = 70; 37.9%). NS: negative symptoms.

Among an initial selection of 88,798 subjects ≥18 years, 1,305 presented a diagnosis of schizophrenia and 1,120 patients were finally analysed. Forty-five patients were excluded as a result of loss of follow-up and 37 because they moved to other areas (Figure 1). Mean age was 46.8 (SD: 13.8) years and 58.4% were male.

Patients were distributed into two groups according to the presence or absence of negative symptoms: group 1 for patients presenting one or more negative symptoms and group 2 for patients without negative symptoms. A 52.5% of patients were allocated to group 1 (N = 588; 95% CI: 49.6-55.4%). We found a greater proportion of male patients in group 1 compared to group 2 (67.7% and 52.0%, respectively; p < 0.001). Dyslipidemia (48.7%), arterial hypertension (38.2%), and diabetes mellitus (19.3%) were the most frequent comorbid conditions. The total mean number of comorbid diagnoses was greater in group 1 than group 2 (8.5 and 7.0, respectively; p < 0.001). Major socio-demographic and clinical characteristics of the patients are shown in Table 2.

**Table 2:** **Socio-demographic and clinical characteristics**

	**Group 1**	**Group 2**	**Total**	**p value**
**Number of patients, %**	**N = 588 (52.5%)**	**N = 532 (47.5%)**	**N = 1,120 (100%)**	
*Socio-demographic characteristics*				
Mean age, years	47.3 (12.2)	46.5 (14.8)	46.8 (13.8)	0.351
Ranges: 18–44 years	40.0%	47.9%	44.6%	
45-64 years	57.0%	43.2%	48.8%	
65-74 years	2.8%	8.6%	6.3%	
≥75 years	0.2%	0.3%	0.3%	<0.001
Gender, male	67.7%	52.0%	58.4%	<0.001
Occupational status, retired	10.4%	10.8%	10.7%	0.662
*Overall comorbidity*				
Mean number of comorbidities	8.5 (4.7)	7.0 (4.1)	7.6 (4.4)	<0.001
Mean Charlson Index	0.5 (0.8)	0.4 (0.7)	0.5 (0.8)	0.130
Mean RUBs	3.1 (0.6)	3.0 (0.7)	3.0 (0.7)	0.098
1 (very low)	1.2%	3.4%	2.5%	
2 (low)	11.4%	11.8%	11.7%	
3 (moderate)	69.6%	68.9%	69.2%	
4 (high)	15.2%	13.6%	14.3%	
5 (very high)	2.6%	2.3%	2.4%	0.218
*Comorbid diagnoses*				
Arterial hypertension	44.1%	34.1%	38.2%	0.001
Diabetes mellitus	22.5%	17.1%	19.3%	0.024
Dyslipidemia	54.1%	44.9%	48.7%	0.002
Obesity	28.4%	18.7%	22.7%	0.001
Vascular events	19.0%	16.3%	17.4%	0.245
Organ failure	17.9%	15.3%	16.3%	0.239
Bronchial asthma	8.7%	5.6%	6.9%	0.041
COPD	5.9%	3.6%	4.6%	0.073
Neuropathies	3.9%	3.0%	3.4%	0.409
*Risk factors*				
Active smoking	28.4%	30.5%	29.6%	0.443
Alcoholism	7.2%	5.4%	6.2%	0.227
Mean time since schizophrenia onset, years	19.9	18.2	18.9	0.093

Group 1: presence of one or more negative symptoms; Group 2: absence of negative symptoms; RUBs: resource utilisation bands; COPD: chronic obstructive pulmonary disease. Values are given as percentage or mean (standard deviation).

Prevalence of individual negative symptoms ranged from 26.1% (G16, active social avoidance) to 60.5% (N4, passive/apathetic social withdrawal). The number of negative components per patient was as follows: 1 (30.1%), 2 (46.5%), 3 (12.5%), 4 (8.5%) and 5 (6.3%). Distribution of symptoms is described in Table 3. In the binary correlation model, N1-blunted affect showed moderate association with N3-poor rapport (r = 0.553) and N4-social withdrawal (r = 0.501), p < 0.001. The same was found between N6-lack of spontaneity and conversation flow and N3 (r = 0.327), p < 0.001.

**Table 3:** **Distribution of negative symptoms**

**Individual PANSS NSFS items**	**Prevalence (%)**
N1 Blunted affect	40.4
N2 Emotional withdrawal	49.8
N3 Poor rapport	41.5
N4 Passive/apathetic social withdrawal	60.5
N6 Lack of spontaneity/conversation flow	22.3
G7 Motor retardation	29.5
G16 Active social avoidance	26.1
Number of negative symptoms	Prevalence (%)
1	30.1
2	46.5
3	12.5
4	8.5
5	6.3

NSFS: Marder Negative Symptoms Factor Score [20].

Quetiapine (32.3%), risperidone (20.3%) and olanzapine (16.4%) were the most common antipsychotic drugs administered. Polypharmacy was common. Mean number of drugs per patient was 3.1 (SD: 1.9) and was found to be significantly greater in group 1 (3.6 vs. 2.6, for group 1 and 2 respectively; p = 0.002). More patients were receiving antidepressive drugs and sedative/hypnotic drugs in group 1 than 2: 88.4% vs. 70.8%; p < 0.001 and 85.3% vs. 71.2%; p < 0.001, respectively.

In the logistic model, the presence of negative symptoms was associated with being male (OR = 1.7; 95% CI: 1.3-2.3), dyslipidemia (OR = 1.4; 95% CI: 1.2-1.7), obesity (OR = 1.4; 95% CI: 1.1-1.9) and arterial hypertension (OR = 1.2; 95% CI: 1.1-1.8); p < 0.05.

Table 4 describes the use of healthcare and non-healthcare resources by each study group. Patients with negative symptoms used more healthcare resources, especially with regard to primary care visits (16.6 vs. 13.9 for group 1 and 2, respectively; p = 0.001). The cost for all study patients was 2.1 million euros, 92.4% of which were healthcare costs and 7.6% indirect costs (productivity losses). Of the total healthcare costs, 77.2% were incurred in primary care and 15.1% in specialized care. Pharmacological treatment was the largest component of the total cost (45.5%). The average/unit cost of schizophrenia was €1,930.80. The average/unit healthcare cost of subjects with negative symptoms was €2,170.00 vs. €1,765.30 for group 1 and 2 respectively, p < 0.001. The average/unit cost corrected for covariables (ANCOVA) in the presence/absence of negative symptoms was €2,190.80 vs. €1,787.60, p < 0.001; and the healthcare cost was €2,085.00 vs. €1,659.10, p < 0.001, respectively. The total cost of subjects with ≤ 2 versus ≥ 3 negative components was similar (€2,190.3 and €2,102.4, respectively; p = 0.883).

**Table 4:** **Resource use and costs**

**Study groups**	**Group 1**	**Group 2**	**Total**	**p value**
**Number of patients, %**	**N = 588 (52.5%)**	**N = 532 (47.5%)**	**N = 1,120 (100%)**	
*Use of resources*				
Primary care visits	16.6 (14.0)	13.9 (13.7)	15.0 (13.9)	0.001
Lab tests	5.3 (3.8)	5.3 (4.8)	5.3 (4.4)	0.858
Conventional radiology	2.5 (2.7)	2.1 (2.4)	2.3 (2.5)	0.003
Supplementary tests	1.2 (1.6)	1.0 (2.0)	1.1 (1.9)	0.098
Specialized care visits	2.6 (4.6)	2.1 (4.4)	2.3 (4.5)	0.076
Emergency room	0.4 (0.9)	0.4 (0.8)	0.4 (0.9)	0.454
Work productivity losses, days	1.6 (12.7)	1.4 (9.7)	1.5 (11.0)	0.741
*Uncorrected cost model*				
Healthcare costs	2,009.1 (1,526.1)	1,626.9 (1,071.6)	1,783.2 (1,290.3)	<0.001
General care	1,684.9 (1,325.5)	1,357.7 (858.5)	1,491.5 (1,085.7)	<0.001
Medical visits	385.0 (324.5)	322.8 (318.8)	348.3 (322.5)	0.001
Lab tests	119.2 (85.6)	118.2 (106.1)	118.6 (98.2)	0.858
Conventional radiology	47.0 (50.4)	38.4 (43.9)	41.9 (46.8)	0.003
Supplementary tests	114.3 (158.5)	96.1 (195.1)	103.5 (181.2)	0.098
Medication	1,019.4 (1,247.5)	782.2 (747.2)	879.2 (989.4)	<0.001
Specialized care	324.2 (503.4)	269.2 (477.0)	291.7 (488.5)	0.064
Medical visits	272.9 (480.6)	222.5 (457.4)	243.1 (467.5)	0.076
Emergency room	51.3 (105.2)	46.7 (99.3)	48.6 (101.8)	0.454
Non-healthcare costs (productivity)	160.9 (1,288.9)	138.4 (983.2)	147.6 (1,117.9)	0.741
Total costs	2,170.0 (1,955.4)	1,765.3 (1,466.6)	1,930.8 (1,694.6)	<0.001
Corrected cost model*			*Difference*	
Healthcare costs	2.085.0	1659.1	426	<0.001
95% CI	1,961.6 - 2,208.5	1,562.6 - 1,755.6		
General care	1,746.4	1374.4	372	<0.001
95% CI	1,640.9 - 1,851.9	1,291.9 - 1,456.8		
Specialized care	338.7	284.7	54	0.084
95% CI	290.5 - 386.8	247.1 - 322.4		
Non-healthcare costs (productivity)	128.5	105.8	23	0.741
95% CI	1.1 - 208.4	45.6 - 211.3		
Total costs	2,190.8	1,787.6	403	<0.001
95% CI	2,026.8 - 2,354.8	1,659.3 - 1,915.8		

Group 1: presence of one or more negative symptoms; Group 2: absence of negative symptoms; CI: confidence interval. Values are given as percentage or mean (standard deviation). *ANCOVA model: tests based on comparison of pairs, linearly independent, between estimated marginal means.

## Discussion

Negative symptomatology is a target of increasing interest. These types of symptoms of schizophrenia are better predictors of functioning than positive symptoms and often associated with a poor response to available pharmacological treatments [5,7,27].

Our study showed the high prevalence of these symptoms and the impact on the economic burden. Using electronic medical records from a population database, one or more negative symptoms were found in 52.5% of patients. The most frequent symptoms were passive/apathetic social withdrawal and emotional withdrawal. Presence of negative symptomatology was associated with maleness and somatic comorbidity. A previous cross-sectional study in Spain also found a high frequency of negative symptoms (57.6%) in a sample of adult outpatients with schizophrenia receiving oral antipsychotic treatment [4]. In addition, social withdrawal and emotional withdrawal were also the most common symptoms (45.8% and 39.1%, respectively).

Research on the costs of care for patients suffering with schizophrenia has increased during recent years [12]. However, comparing costs between countries with different socioeconomic, cultural, epidemiological background and different systems for organizing and funding health care is very difficult [12,14]. In Spain, the available data for cost of illness of schizophrenia is limited [13,15]. One study estimated the total costs of schizophrenia to be €1,970.8 million (direct medical costs accounting for 53% and informal care costs, 47%) [13]. Despite having implemented a conservative approach, the health care costs associated with schizophrenia account for 2.7% of total public health care expenditure in Spain [13]. In addition, the impact of negative symptoms on schizophrenia related to healthcare costs evidence is still scarce.

A cluster analysis that identified patients with similar profiles of symptoms found that monthly treatment costs were higher when predominant negative symptoms were presented compared with low or moderate negative symptoms [16]. The Epsilon study conducted in five European countries estimated an association between direct costs and negative symptoms with its limitations [17,28]. The impact of negative symptoms on service costs (inpatient care, outpatient care, day care, community services, residential care, all services) was measured. Results differed between countries due to site specific differences in clinical characteristics, service use and costs. In our study, the adjusted unit healthcare cost related to the presence/absence of negative symptoms was €2,190.80 and €1,787.60 and the healthcare cost was €2,085.00 and €1,659.10, respectively; (p < 0.001). Patients with negative symptoms used more healthcare resources over a period of 12 months, especially regarding to primary care assistance. All these findings support that the presence of negative symptoms can increase the costs of schizophrenia.

Our study has several limitations inherent to research based on population databases [29]. PANSS scale is not routinely completed in mental health services. Collecting negative symptoms from open text recorded in the medical records may be associated with an underestimation of the true prevalence of these symptoms in patients with schizophrenia and their real impact in terms of costs and utilization of resources due to the under-registration of them by the clinicians. In addition, the most severe cases were possibly not included in the study because they are usually not seen as outpatients. The costs for any side-effects of antipsychotic drugs were neither analyzed. The only direct costs considered were those relating to the public health system and the area of influence of the patient. Sick leaves (temporary or permanent) may in turn be a limited indicator of indirect costs because it was not considered premature death and it was not considered the informal costs. Despite these limitations, the results found herein reflect the study’s strength of comparing the economic impact of the disease differentiating between the presence or absence of negative symptoms.

Negative symptoms are still considered controversial because of the difficulty to define and measure them as well as designing specific clinical trials [30,31]. Unfortunately, with the exception of amisulpride in some European countries, there are no pharmacological agents approved for the treatment of negative symptoms [6].

## Conclusion

The social and economic costs of schizophrenia are considerable. The prevalence of negative symptoms among patients with schizophrenia receiving antipsychotic treatments is high and associated with a relevant economic impact on the healthcare system.

Further studies, including new specific assessment scales, are necessary to determine the association between the presence of negative symptoms and greater healthcare resource utilization, predisposing factors, and underlying mechanisms.
